# Mechanical properties of pediatric low-grade gliomas in children with and without neurofibromatosis type 1

**DOI:** 10.1007/s00234-024-03491-z

**Published:** 2024-10-21

**Authors:** Grace McIlvain, Laura L. Hayes, Andrew W. Walter, Lauren W. Averill, Vinay Kandula, Curtis L. Johnson, Rahul M. Nikam

**Affiliations:** 1 Department of Biomedical Engineering, Columbia University, New York, NY USA; 2 Department of Radiology, Columbia University, New York, NY USA; 3 Department of Radiology, Nemours Children’s Hospital, Orlando, FL USA; 4 Department of Hematology/Oncology, Nemours Children’s Hospital, Wilmington, DE USA; 5 Department of Radiology, Nemours Children’s Hospital, Wilmington, DE USA; 6 Department of Biomedical Engineering, University of Delaware, Newark, DE USA

**Keywords:** Pediatric low-grade gliomas, Neurofibromatosis type 1, Focal abnormal signal intensities, Magnetic resonance elastography, Mechanical properties

## Abstract

**Introduction:**

Prognoses for pediatric brain tumors are suboptimal, as even in low-grade tumors, management techniques can lead to damage in the developing brain. Therefore, advanced neuroimaging methods are critical for developing optimal management plans and improving patient care. Magnetic resonance elastography (MRE) has allowed for the characterization of adult gliomas by their mechanical properties, which are uniquely sensitive to the complex interplay of cellularity, vasculature, and interstitium. However, pediatric tumors differ in behavior and cytoarchitecture, and their mechanical properties have never been assessed.

**Methods:**

Here, we conduct the first study of pediatric brain tumor mechanical properties by using MRE to measure tissue stiffness and damping ratio in low grade gliomas (LGGs). We additionally measure the mechanical properties of non-neoplastic focal abnormal signal intensities (FASIs) in children with neurofibromatosis type 1 (NF1).

**Results:**

23 patients age 4–17 years who had MR imaging results consistent with a primary LGG or with NF1 were included in this study. We found that pediatric gliomas are on an average 10.9% softer (*p* = 0.010) with a 17.3% lower (*p* = 0.009) viscosity than reference tissue. Softness of tumors appeared consistent across tumor subtypes and unrelated to tumor size or contrast-enhancement. In NF1 we found that, unlike gliomas, FASIs are stiffer, though not significantly, than reference tissue by an average of 10.4% and have a 16.7% lower damping ratio.

**Conclusions:**

Measuring tumor mechanical properties patterning and heterogeneity has potential to aid in prediction of biological behavior and inform management strategies for pediatric patients.

## Introduction

Low grade gliomas (LGGs) are characterized by slow growth rates and relatively favorable prognosis compared to higher grade brain tumors [1]. The mainstay of LGG therapy is surgical excision, which can be curative with total resection [2]; patients who do not undergo a complete resection are often treated with standard chemotherapy or radiation therapy and have a 10-year progression-free survival rate of greater than 80%^3^. Management techniques for adults with LGGs have improved to the point of making some LGGs low risk; however, *pediatric* LGGs require unique considerations.

Pediatric tumors differ in cytoarchitecture and behavior from adult tumors. Uniquely, pediatric LGGs rarely show malignant progression [3] and rarely have *IDH* mutations [4, 5]. Despite these favorable conditions, prognoses for pediatric tumors remain suboptimal, as LGGs can demonstrate infiltrating behavior that affect critical neuroanatomical structures, and management techniques including surgical resection, chemotherapy, and radiation lead to tissue damage in the developing brain [6]. Therefore, strategies for clinical management of LGGs must be weighed carefully in the pediatric brain to consider a patient’s holistic health and development.

A major challenge in evaluating pediatric gliomas is that clinical presentations vary widely and are dependent in part on the size, location, shape, and interactions of the tumor with surrounding tissues [4]. While MRI is valuable for identifying abnormalities, it does not provide complete characterization alone. For instance, in LGGs, contrast enhancement, decreased diffusivity, edema, and necrosis, suggest a more aggressive tumor, but these features are not necessarily grade specific [7]. Even when using invasive methods like biopsies, tumor heterogeneity poses limitations for obtaining sufficient sampling across the entire tumor. Therefore, novel imaging modalities which can noninvasively describe a unique aspect of underlying tissue cellularity warrant investigation for characterizing pediatric LGGs.

Magnetic resonance elastography (MRE) has emerged in the last decade as a leading method for in vivo quantification of brain mechanical properties [8]. MRE is a phase-contrast MRI technique which uses shear wave vibrations to characterize tissue stiffness and viscosity. MRE has demonstrated high efficacy for sensitive characterization of tissue microstructure and has recently become the gold standard for staging liver fibrosis, proving better even than liver biopsy, as it is noninvasive and can capture the entire tissue area in a single scan [9]. The mechanical properties of tumors and other lesions are thought to be uniquely sensitive to the complex interplay of tissue cellularity, vasculature, and interstitium [10]. Mechanical properties describe tissue microstructural cohesiveness; tissues with dense, well unified, well contained cells, that have strong structural cohesion, tend to have higher stiffness and lower viscosity [11]. MRE has been used in presurgical characterization of brain tumors in adults, particularly in determining if meningiomas are stiff or soft to inform surgical resection planning [12–14] and to demonstrate that adult gliomas are softer and have lower viscosity than reference tissue [15–18].

While most gliomas have no known cause, approximately 20% of pediatric gliomas occur in individuals with genetic predispositions for glioma development [3]. Neurofibromatosis type 1 (NF1), for example, is an autosomal dominant condition resulting in varied central nervous system manifestations including optic pathway gliomas and white matter changes [19]. These white matter changes, called focal abnormal signal intensities (FASIs) [19], are non-neoplastic small hyperintensities which present typically in the basal ganglia or cerebellar regions. FASIs are primarily identified in childhood [20], and their presence, severity, and microstructural composition is not well understood. Mechanical properties offer a unique image modality which can be used to noninvasively characterize FASI and differentiate them from healthy tissue, as well as LGGs also developing in patients with NF1.

To date, no study has used MRE to non-invasively assess the mechanical properties of pediatric gliomas, and in general, LGGs have been mostly overlooked. Here we investigate the mechanical properties of pediatric central nervous system lesion and consider the spatial heterogeneity of tumor mechanics, the variability between tumor types, and the exclusivity from other imaging contrasts.

## Methods

This study was approved by the Institutional Review Board of Nemours Children’s Health System and all parents or guardians of participants gave informed written consent. *Inclusion Criteria*: Patients aged 3–17 years that had MRI imaging consistent with a diagnosis of either a primary LGG or NF1. *Exclusion criteria*: Adults (> 18 years); patients receiving radiotherapy, chemotherapy, or have previously had a surgical resection; patients with tumors near high-susceptibility regions (e.g. paranasal sinuses); patients with MRI corrupted by significant metal-related artifacts; medically vulnerable populations.

Patients underwent both standard-of-care clinical MR imaging and a research MR elastography scan on a GE Signa 3T PET/MR scanner (GE Healthcare; Waukesha, WI). Patients received all sequences consistent with standard-of-care imaging for their diagnosis, including but not limited to a sagittal T_2_-weighted 3D fast spin echo (CUBE sequence) with axial and coronal reconstructions, axial FLAIR (Fluid Attenuated Inversion Recovery), sagittal pre- and post-contrast T_1_-weighted BRAVO (Brain Volume Imaging; Ultrafast GRE sequence) with axial and coronal reconstructions, and axial pre- and post-contrast sagittal T_1_- MEMP (Multi Echo Multiplanar) fat saturated spin-echo imaging.

The MRE images were acquired using a single-shot 2D-EPI sequence at 2.5 mm isotropic resolution, with FOV of 240 × 240 mm [2], matrix size 96 × 96, 48 slices, and TR/TE of 7200/88 ms. Vibrations were delivered at 50 Hz to the head using a passive-driver head pillow connected to a pneumatic actuator (Resoundant; Rochester, MN), with low vibrational amplitudes that were suitable for children. Motion encoding gradients at 40 mT/m were synchronized with applied motion to encode tissue displacement into the phase image. Motion encoding gradients were applied in each of the primary orthogonal directions and for each direction four time points were acquired by changing the synchronization over one period of vibration. The MRE imaging time was 3 min 28 s.

MRE data were unwrapped using FSL PRELUDE [21] and processed to generate 3D, full vector, complex displacement fields across the entire brain. Displacement images were used with a nonlinear inversion algorithm (NLI) [22] to estimate brain viscoelastic properties. NLI returns whole brain maps of the complex shear modulus, $G^{*}=G'+iG''$, which is converted to viscoelastic shear stiffness, $\mu=2|G^{*}|^{2}/(G'+|G^{*}|)$, and damping ratio, $\xi = G{^{\prime \prime}}/2G{^{\prime}}$.

Quality of displacement maps was confirmed for all patients through the octahedral-shear strain signal-to-noise ratio [23], which is a standard MRE measure of signal-to-noise ratio, with values greater than 3.0 being sufficient for robust calculation of mechanical property maps with NLI.

Regions which were indicated with high certainty as gliomas were manually segmented under the guidance of a pediatric neuroradiologist (RMN or LLH) on the T_2_-weighted images, while referencing the additional scans as needed. Size of the apparent gliomas was defined by the longest points in each of the primary orthogonal directions on imaging, and by calculating the volume of the three-dimensional segmented region. Determination of if a region was contrast enhancing was made by the neuroradiologist from the T_1_-weighted post-contrast BRAVO images. Tumor grade was determined through pathology in cases where tissue samples were obtained.

Regions which appeared as small white matter hyperintensities on FLAIR, clustered in conventional locations, and indicated as FASIs consistent with NF1 during a clinical read, were included. These regions were manually segmented under the guidance of a pediatric neuroradiologist (RMN or LLH). When apparent FASIs were small and clustered closer than 4 mm, the region of interest was drawn around the entire area.

Reference tissue for each patient was chosen as the average of all normal appearing white matter in both the cerebrum and cerebellum that was further than 12 mm away from the tumor or FASI boundary. Mechanical properties were calculated by registering the tumor or FASI segmentation masks to the MRE images using FSL FLIRT [21], and multiplying the mask of the segmented region by the maps of stiffness and damping ratio and calculating the average value across the spatial area. Region heterogeneity was calculated by the standard deviation of stiffness or damping ratio across the spatial area. Paired t-tests were used to calculate differences between tumor or FASI and reference tissue.

## Results

### Patient Population

Twenty-three pediatric patients ages 4–17 years (mean age: 10.3 years; 14 female, 9 male) with imaging consistent with a either a primary LGG or NF1 underwent both standard-of-care clinical MR imaging and a research MR elastography scan. In total we collected data on 20 LGGs, from 17 patients (9 Female, 8 Male) ages 5–16 (mean age: 10.9 years), and 17 FASI areas from 7 patients with NF1 (6 Female, 1 Male) ages 4–17 years (mean age: 9.9 years). One patient was categorized as having both NF1 and primary LGG (Patient 6). Patient demographics are described in Table 1.

**Table 1:** Demographics of pediatric low-grade glioma patients

ID	Age (Years)	Sex	Location		Grade	# of ROIs	Size	Characterization	NF1?
*Low Grade Glioma*									
1	16.5	F	Trigone of the left lateral ventricle		I	1	3332 cm^3^	Pilocytic astrocytoma	No
2	9.5	F	Left temporal lobe		I	1	262 cm^3^	Dysembryoplastic neuroepithelial tumor	No
3	6.9	M	Suprasellar, hypothalamic		I	2	2147 cm^3^ 3375 cm^3^	Ganglioglioma	No
4	14.5	M	Medial anterior left cerebellum		II	1	578 cm^3^	Diffuse astrocytoma	No
5	7.7	F	Medial left temporal lobe		I	1	201 cm^3^	Low grade glioma	No
6	16.6	F	Dorsal medulla mass		n/a	1	717 cm^3^	Pontine glioma	Yes
7	14.5	M	Optic chiasm/hypothalamus		I	2	3694 cm^3^ 904 cm^3^	Multilobulated mass corresponding to a pilocytic astrocytoma, with multiple cystic components	No
8	13.0	F	Optic chiasm, prechiasmatic optic nerves		I	1	1978 cm^3^	Optico-chiasmatic pilocytic astrocytoma	No
9	8.1	F	Hypothalamus/right parietal lobe		I	2	5867 cm^3^ 12,859 cm^3^	Optico-chiasmatic pilocytic astrocytoma	No
10	7.9	M	Right cerebellar vermis		n/a	1	648 cm^3^	Cerebellar glioma	No
11	11.1	M	Left postcentral gyrus		n/a	1	693 cm^3^	T2 hyperintense, T1 hypointense nodule	No
12	8.5	F	Left middle cerebellar peduncle		n/a	1	153 cm^3^	Lesion consistent with low-grade glioma	No
13	13.9	F	Left medial cerebellar hemisphere		n/a	1	605 cm^3^	Low Grade Glioma	Yes
14	12.5	M	Centered in the left basal ganglia and hypothalamus		II	1	5077 cm^3^	Cystic mass lesion consistent with low-grade glioma	No
15	10.7	M	Centered in the suprasellar region		II	1	1654 cm^3^	Suprasellar pilomyxoid astrocytoma,	No
16	8.1	F	Body of the left corpus callosum		n/a	1	517 cm^3^	Low-grade glioma	Yes
17	5.7	M	Right posterior insular cortex		n/a	1	631 cm^3^	Low-grade glioma	Yes
				*Focal Abnormal Signal Intensities*					
6	16.6	F	Periventricular white matter, including the right insular subcortical white matter			1	615 cm^3^	FASIs	Yes
18	10.7	F	Right greater than left basal ganglia and periventricular white matter bilaterally			3	646 cm^3^ 7460 cm^3^ * 9756 cm^3^ *	FASIs	Yes
19	4.8	F	Bilateral globi pallidi, thalami, bilateral deep cerebellar white matter			3	343 cm^3^ 526 cm^3^ 71 cm^3^	FASIs	Yes
20	11.9	F	Bilateral basal ganglia, and bilateral cerebellar white matter.			2	507 cm^3^ 615 cm^3^	FASIs	Yes
21	9.0	F	Right greater than left globi pallidi, left thalamus, and bilateral cerebellar white matter			3	720 cm^3^ 743 cm^3^ 448 cm^3^	FASIs	Yes
22	11.8	F	Bilateral globi pallidi, right posteromedial temporal lobe white matter, posteromedial right temporal lobe			3	281 cm^3^ 393 cm^3^ 368 cm^3^	FASIs	Yes
23	4.6	M	Supratentorial and infratentorial deep white matter; right medial cerebellar hemisphere			2	167 cm^3^ 620 cm^3^	FASIs	Yes

Abbreviations: NF1 – Neurofibromatosis Type 1; M– Male; F– Female

*Encompassing FASIs clustered closer than 4 mm within the same local region

### Low grade gliomas

**Fig. 1:**
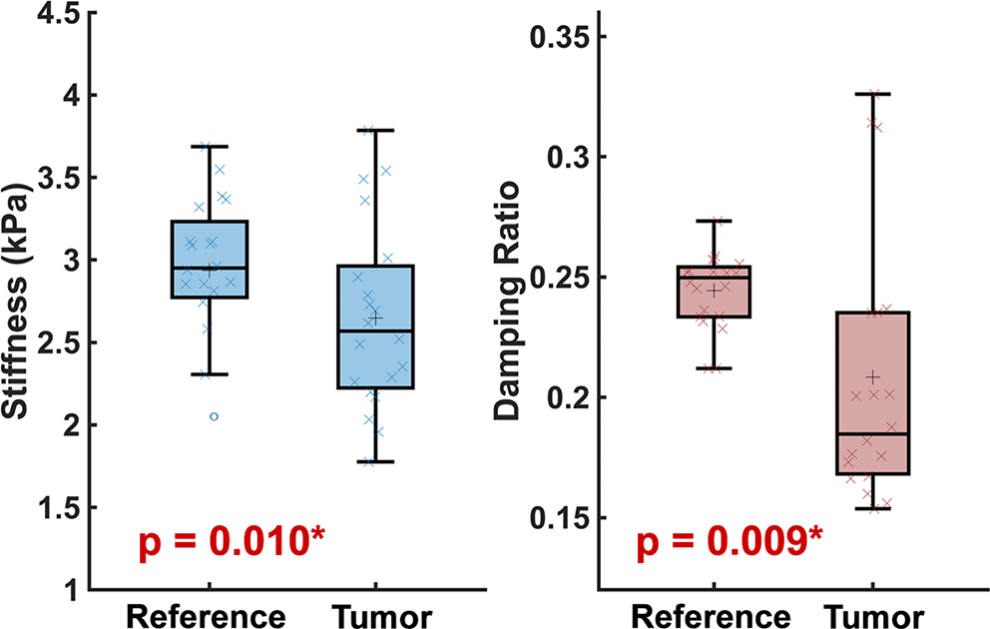
Stiffness (kPa), $\mu$, and damping ratio, $\xi,$ of pediatric low-grade gliomas compared to reference normal appearing white matter measured with magnetic resonance elastography. Low-grade gliomas are significantly softer and have significantly lower damping ratio than reference tissue

We find that on average, stiffness of gliomas measured 2.65 ± 0.56 kPa, while normal-appearing white matter stiffness measured 2.94 ± 0.44 kPa, with average stiffness difference of 10.9% (*p* = 0.010; Fig. 1a). Patient 12, with a middle cerebellar peduncle tumor, had the largest difference in stiffness between tumor (1.95 ± 0.21 kPa) and reference (3.09 ± 0.46 kPa) of 57.7%. The softest tumor was in Patient 7, with a stiffness of 1.77 ± 0.50 kPa; however, Patient 7 also had soft reference tissue of 2.05 ± 0.57 kPa, and as such the tumor stiffness difference was 17.6%. Two patients had tumors that were > 5% stiffer than reference tissue: Patient 5 with an LGG in the medial left temporal lobe, and Patient 10 with a LGG in the right cerebellar vermis.

We also found that tumor damping ratio was reduced compared to reference tissue, with tumor mean damping ratio of 0.208 ± 0.055 and reference white matter damping ratio of 0.244 ± 0.016 (*p* = 0.009; Fig. 1b). Tumor damping ratio ranged from a lowest value of 0.154 ± 0.057 in Patient 14 (77.7% lower than reference), to a highest damping ratio of 0.326 ± 0.070 in Patient 17 (28.3% higher than reference). Fifteen tumors had lower damping ratio than reference tissue, while only three patients had tumors with damping ratio > 5% higher than the reference region.

**Fig. 2:**
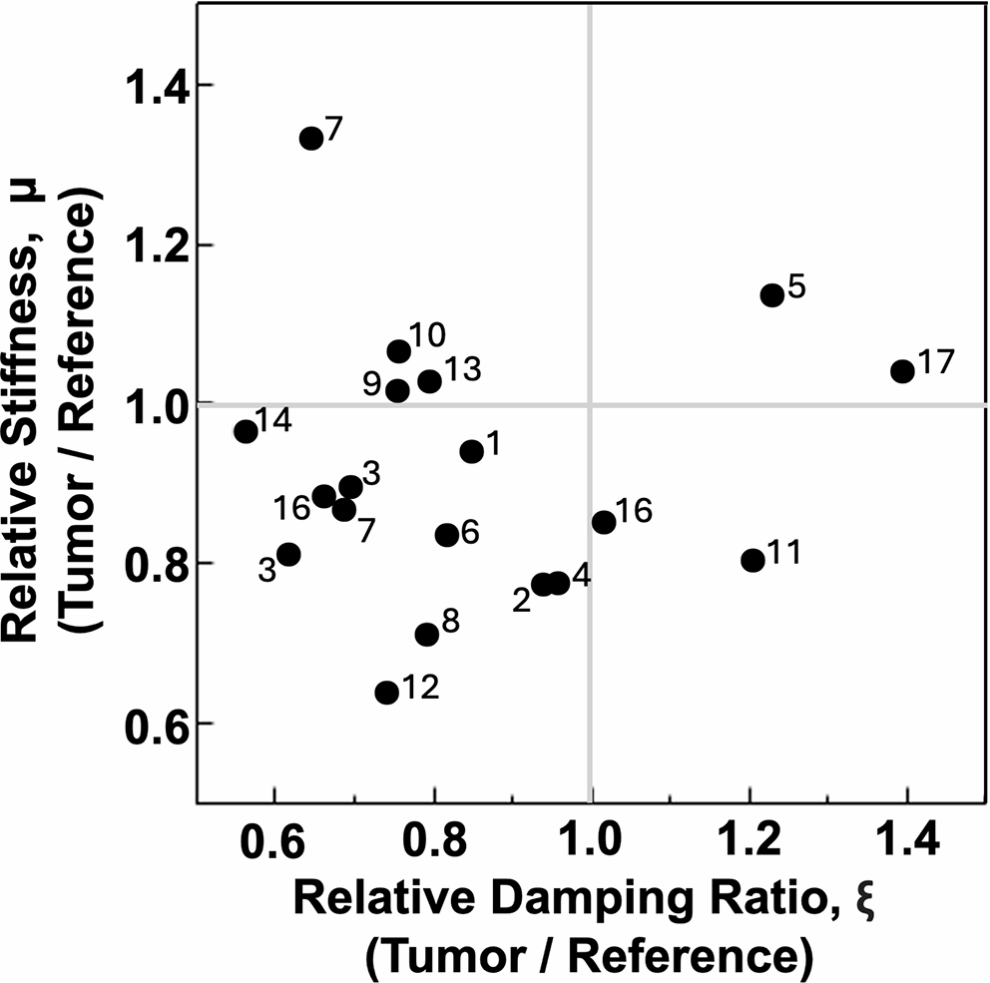
Mechanical properties of each pediatric low-grade glioma shown as relative stiffness ($\mu$) and relative damping ratio ($\:\varvec{\xi\:}$) of the low-grade glioma, both compared to the reference white matter. Numbers indicate patient ID number found in Table 1. Four tumors exhibited either stiffness or damping ratio or both that were > 5% greater than the reference tissue

Figure 2 plots the relative stiffness and damping ratio of each tumor compared to reference tissue. Most tumors had both lower stiffness and lower damping ratio, while only four of the tumors exhibited either stiffness or damping ratio or both that were meaningfully higher than the reference.

**Fig. 3:**
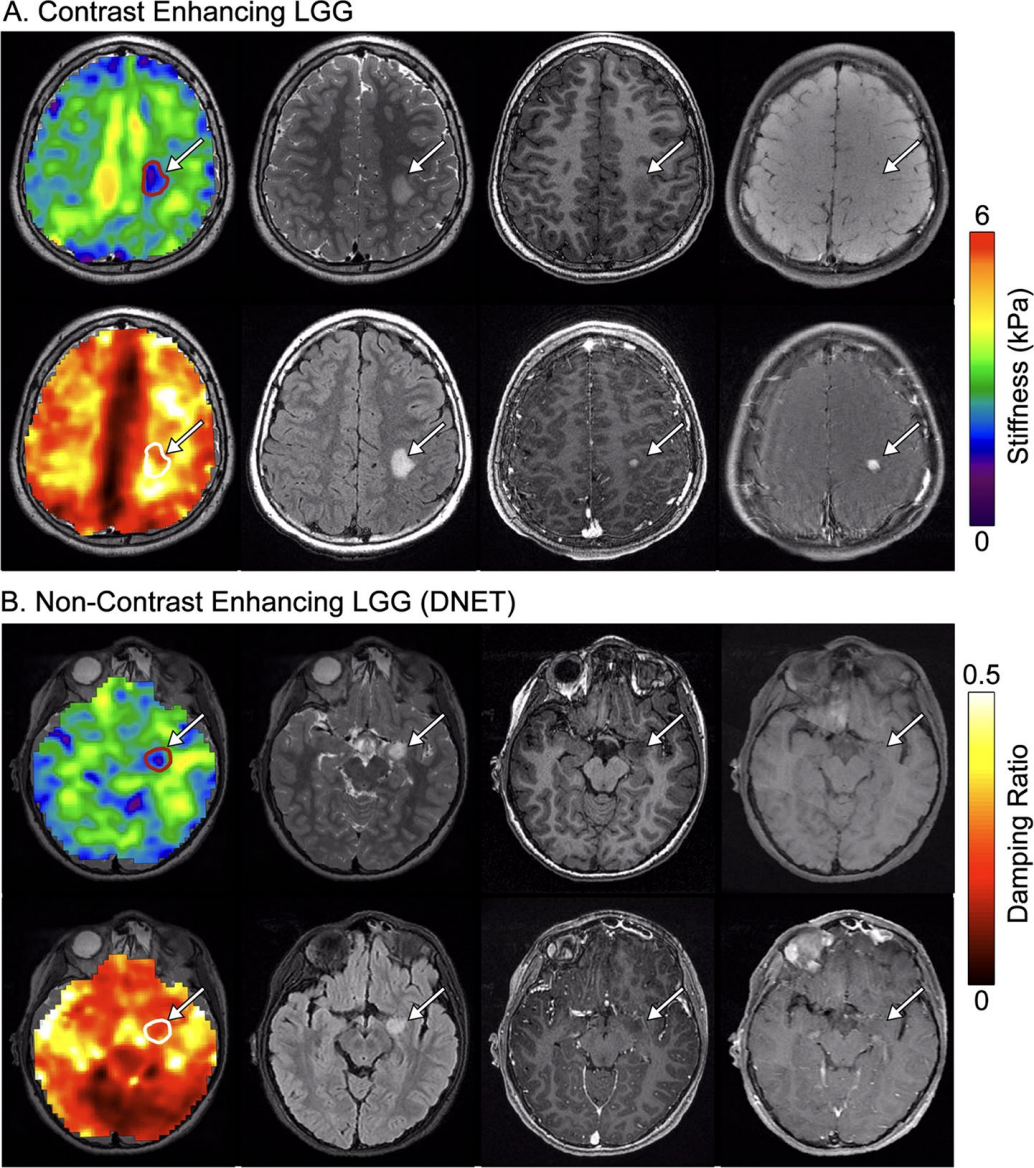
Both contrast enhancing and non-contrast enhancing LGGs appear soft. (**A**) Patient 11, 11-year-old male, with a contrast enhancing low-grade glioma and focal seizures. (**B**) Patient 2, 9-year-old female, with a non-contrast enhancing left temporal lobe dysembryoplastic neuroepithelial tumor (DNET). Top rows, left-to-right: Stiffness map, T_2_-CUBE, T_1_-BRAVO, MEMP; Bottom rows, left-to-right: Damping ratio map, T_2_-FLAIR, post contrast T_1_-BRAVO, post contrast MEMP

Stiffness and damping ratio of the tumors appeared consistent across tumor types and unrelated to the presence or lack of contrast enhancement on T_1_ imaging. For example, Fig. 3a shows images from Patient 11, who has a T_2_-hyperintense, T_1_-hypointense, contrast enhancing LGG centered in the left postcentral gyrus, measuring 9 × 6 × 11 mm [24]. Tumor stiffness is 2.35 ± 0.57 kPa, while reference tissue stiffness is 2.94 ± 0.55 kPa (tumor approximately 20.1% softer than reference). Whereas Fig. 3b shows images from Patient 2, who has a T_2_-hyperintense, T_1_-hypointense, non-enhancing dysembryoplastic neuroepithelial tumor (DNET) located in the left temporal lobe, measuring 7 × 7 × 8 mm [24]. Tumor stiffness is 2.17 ± 0.43 kPa, while reference tissue is 2.81 ± 0.56 kPa (tumor 22.5% softer than reference).

Tumors of Patients 11 and 2 are similar in size and conventional MR imaging appearance except for differences in enhancement. Their stiffness measurements are similar, in both absolute value and percent difference compared to reference. However, the DNET (Patient 2) has lower damping ratio of 0.236 ± 0.056 relative to reference tissue of 0.251 ± 0.092, while the contrast-enhancing LGG (Patient 11) has a higher damping ratio at 0.312 ± 0.059 relative to reference tissue of 0.259 ± 0.098.

**Fig. 4:**
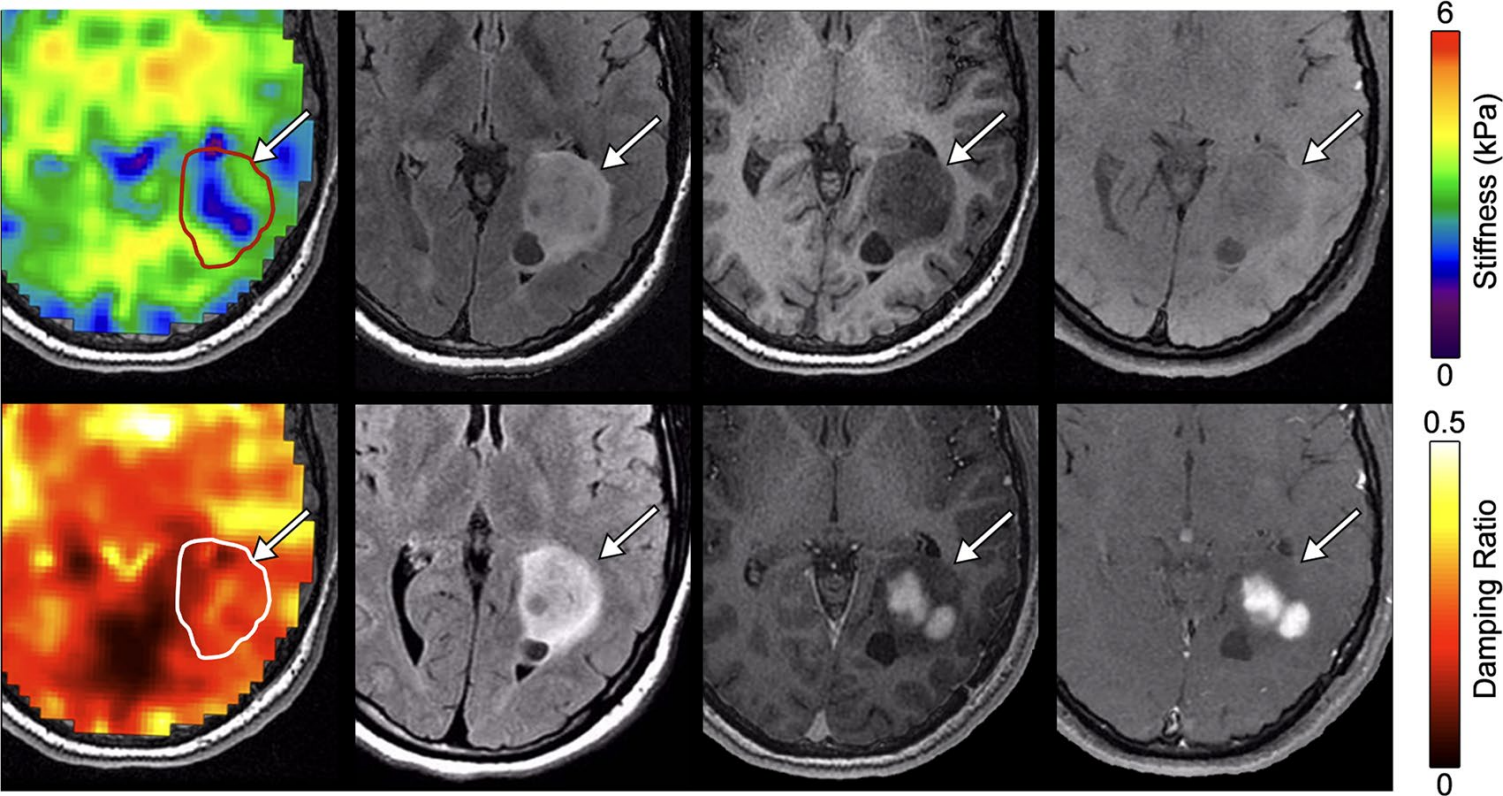
Large low-grade gliomas show mechanical heterogeneity. 16-year-old Female Pilocytic astrocytoma (Patient 1). Top row, left-to-right: Stiffness map, T_2_-CUBE, T_1_-BRAVO, MEMP; Bottom row, left-to-right: Damping ratio map, T_2_-FLAIR, post contrast T_1_-BRAVO, post contrast MEMP

We observed that, in general, larger tumors had more spatial mechanical heterogeneity across the whole lesion. In Patient 1 with a pilocytic astrocytoma in the trigone of the left lateral ventricle (Fig. 4), there was an apparent stiffness standard deviation of 0.78 kPa, which is high compared to stiffness variations in normal appearing white matter (across all patients) of approximately 0.30 kPa. A band of relatively low stiffness tissue can be seen spanning the center of the tumor while the periphery of the tumor appears more similar to reference tissue. This patterning is not observable in the T_1_-weighted, T_2_-weighted, or FLAIR images. In this case, the softening was similar (but not identical) to the pattern of contrast enhancement, though this observation of softening is not consistent across all tumors with partial contrast enhancement.

**Fig. 5:**
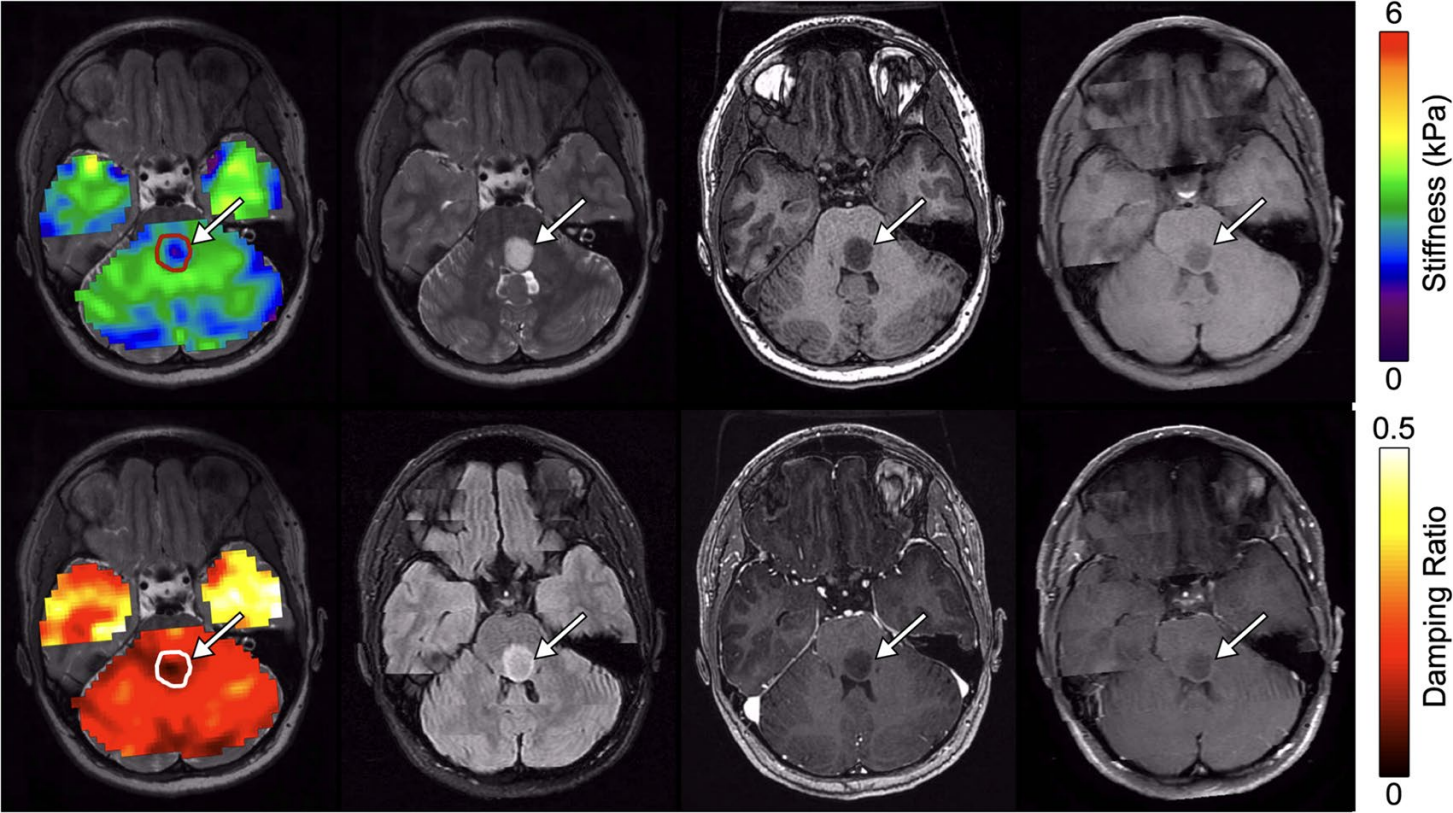
16-year-old female with a pontine glioma and NF1 (Patient 6). Top row, left-to-right: Stiffness map, T_2_-CUBE, T_1_-BRAVO, MEMP; Bottom row, left-to-right: Damping ratio map, T_2_-FLAIR, post contrast T_1_-BRAVO, post contrast MEMP

Our group of patients included seven infratentorial LGGs, and we observed that infratentorial tumors appear to follow the same pattern of mechanical properties as supratentorial tumors. Figure 5 shows an example of a glioma located in the dorsal aspect of the medulla oblongata measuring 11 × 12 × 12 mm [24] (Patient 6). This tumor is soft, at 2.29 ± 0.37 kPa compared to reference tissue of 2.74 ± 0.60 kPa, or 16.4% softer, and has a lower damping ratio of 0.201 ± 0.046 compared reference tissue of 0.246 ± 0.097, an 18.3% difference. Overall, infratentorial LGGs exhibited average stiffness of 2.59 ± 0.73 kPa compared to supratentorial LGGs of 2.69 ± 0.44 kPa (*p* = 0.904) The average damping ratio of infratentorial LGGs was 0.186 ± 0.022 compared to 0.214 ± 0.114 for supratentorial (*p* = 0.217).

### NF1-associated focal abnormal signal intensities

**Fig. 6:**
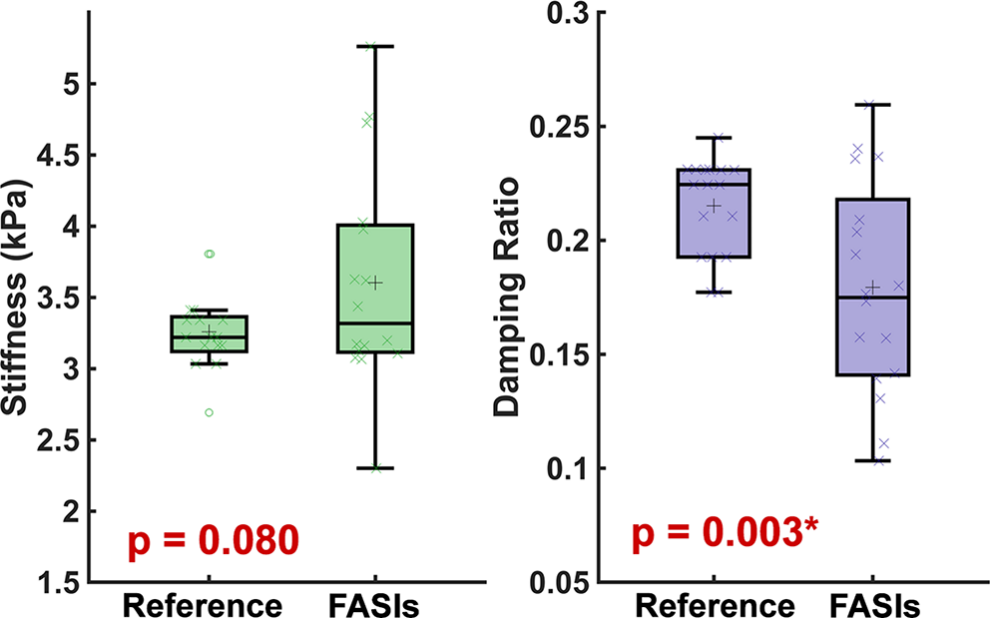
Focal abnormal signal intensities (FASIs) have lower damping ratio than reference tissue in pediatrics with neurofibromatosis type 1 (NF1)

In patients with NF1, we found that FASIs are, on average, slightly stiffer than normal appearing white matter (3.60 kPa vs. 3.26 kPa), though this relationship is not statistically significant (*p* = 0.080; Fig. 6). Interestingly FASIs have a much larger range of stiffness than reference tissue, with some FASIs being as stiff as 5.26 kPa while others are 2.30 kPa, compared to the reference tissue which falls in a range of 3.03 to 3.80 kPa. This equates to some FASIs ranging as high 36.5% stiffer than reference tissue, and as low as 39.9% softer.

**Fig. 7:**
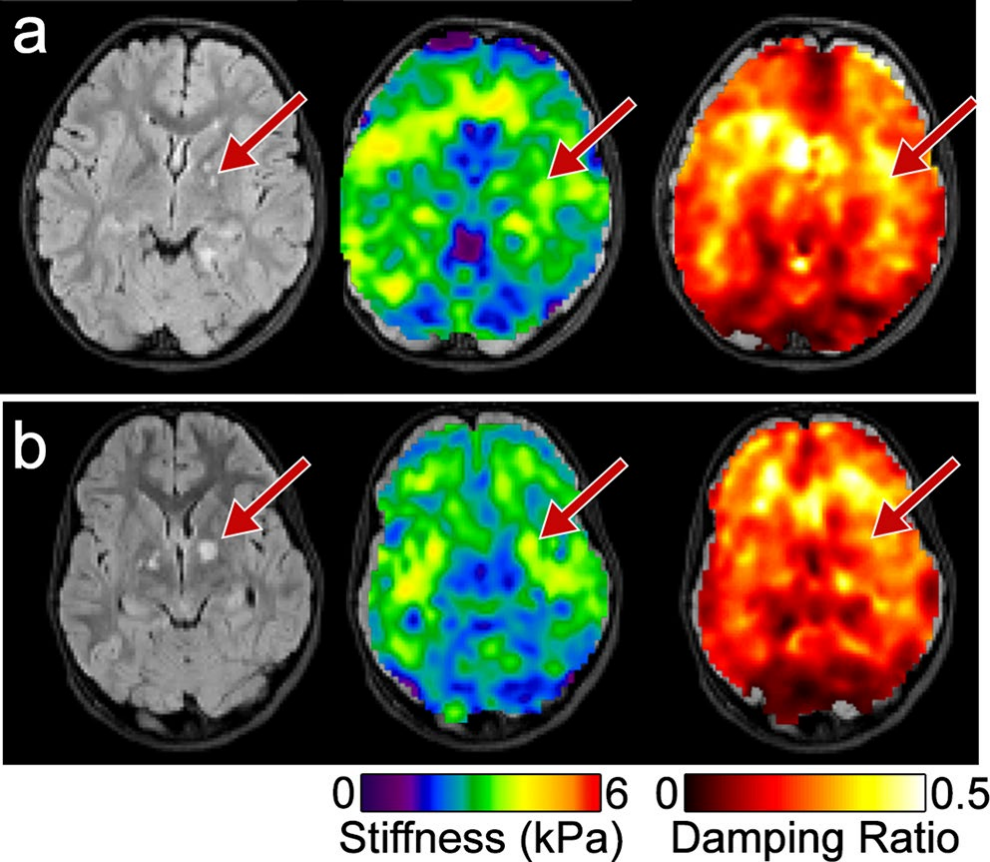
Focal abnormal signal intensities are stiffer than reference white matter tissue, as shown by two pediatric examples. (**A**) 11-year-old female (**B**) 9-year-old female

We also see that FASIs, which conventionally appear bright on a FLAIR image (Fig. 7), are significantly different in damping ratio than normal appearing white matter (*p* = 0.003). Average FASI damping ratio is 0.179 ± 0.047 while reference white matter is 0.215 ± 0.021. All except three FASI areas had damping ratio lower than reference. The lowest damping ratio of any FASI region was 0.103 ± 0.036 in Patient 22, which was 55.4% less than reference of 0.231 ± 0.073. The degree of stiffness or damping ratio difference did not appear to correspond to FASI size or location. When comparing differences in mechanical properties between gliomas and FASIs, relative to normal appearing white matter, we find that FASIs have significantly higher relative stiffness compared to gliomas (*p* = 0.004) but differences in relative damping ratio between FASIs and gliomas was not significant (*p* = 0.804).

## Discussion

To our knowledge, this is the first study utilizing magnetic resonance elastography to non-invasively assess the mechanical properties of pediatric low-grade gliomas. We found that gliomas are significantly softer, and significantly less viscous (lower damping ratio) than normal appearing white matter. We found that the degree of tumor softness appears to be unrelated to tumor size or location, though larger tumors typically show more mechanical heterogeneity than smaller tumors. Interestingly, we also found that in patients with neurofibromatosis type 1, areas of focal abnormal signal intensity have lower damping ratio than the normal appearing white matter.

Our results agree with previous MRE findings in adults, where gliomas are found to be softer than surrounding tissue. Although adult tumor mechanics have been studied, pediatric tumors cannot be assumed to present with the same behavior or hold the same implications without dedicated pediatric research studies. Pediatric gliomas have distinctly different cellular and molecular etiologies than their adult counterparts, leading to differences in tumor location, tumor burden, tumor growth trajectories, and prognoses. In pediatrics, it can be challenging to assess tumor malignancy using a traditional Ki-67 immunostaining, as the developing central nervous system has a naturally robust proliferative potential [3]. Further, glial progenitor cells which serve as precursors for tumors are found in variable abundances in children and adults, and their rates of proliferation vary significantly [25, 26]. These differences in pediatric tumor make it necessary to studying pediatric tumors separately.

In this work we chose study LGGs, as opposed to the more often studied high-grade gliomas, as treatment for LGGs including chemotherapy and radiation can interfere with developmental processes [27]. In children, the decision to intervene in *high*-grade gliomas is much clearer due to the significant, imminent threat they pose, whereas in *low*-grade tumors, if delaying treatment is possible, it may help avoid affects to normal neurodevelopment [27]. Here we find that LGGs are on average ~10% softer than normal appearing white matter. Our study only focuses on LGGs, and therefore we cannot explicitly comment how pediatric glioma mechanical properties are associated with tumor grade, though based on past work, we expect that lower tumor stiffness would occur with increasing grade of pediatric tumors. One adult study showed that Grade II gliomas were ~ 30% softer than reference tissue, while Grade III gliomas are ~ 44% softer than baseline, and Grade IV tumors are ~50% softer than baseline [18]. Our observation of ~10% lower stiffness in Grade I and II tumors in the present study would appear to be consistent with this adult trajectory of grade-dependent softness. Animal models of gliomas have also shown decreasing viscosity across several weeks of tumor progression [28, 29], with a 17% decrease in damping ratio over a four-week period of tumor growth.

It is common practice to consider the mechanical properties of tumors relative to healthy reference tissue, as significant variability in stiffness and damping ratio exists between individuals. Thus, it should not be overlooked that children and adults have fundamental differences in the mechanical properties of their normal brain tissue. Brain maturation between age 5- and 35-years shows decreases in stiffness of approximately 0.3% per year, with a 5-year-old having an average brain stiffness of ~3.2 kPa while a 35-year-old has an average brain stiffness of ~2.9 kPa [30]. Tissue viscosity increases at an approximate rate of 0.4% per year over the same time. While there is no explicit elastography work about the expected alterations to tumor mechanical properties which form in stiffer and softer brains, cytopathology research has repeatedly demonstrated significant differences between the structure and function of cells which were grown on substrates of differing mechanical properties [31].

Despite variations in pediatric and adult cellular markers in tumors, histopathology and immunohistochemistry from adult murine glioma mechanical property studies can allow us to speculate on the implications of our findings [28]. Morphologically, tumors cells are large and amorphous compared to neural cells and have less structural cohesion between cells, making them more soft than healthy neural tissue [32]. Tumors also vary molecularly; one study shows that stiff and soft tumors have different genetic signatures, with genes for matrix reorganization and cellular adhesion being overexpressed in more stiff tumors [33]. More research is needed to fully explain the microstructural changes which portend soft gliomas, but elastography has been demonstrated as a unique tool to investigate these changes [34]. It should be noted, however, that not all brain tumors are soft; for instance, meningiomas are both stiffer and have a higher viscosity than surrounding tissue [13, 28].

In pediatrics, unlike adults, genetic factors such as the presence of NF1 are a primary contributor to the formation of gliomas, and NF1 nearly always shows the presence of non-neoplastic FASIs. As the size and number of FASIs can fluctuate over time, early-stage gliomas can be mistaken for FASIs in patients with NF1. Differentiating FASIs from healthy tissue, and from lesions likely to show radiologic progression, can be the defining factor in developing a suitable tumor monitoring plan. Here, we find that FASIs appear as slightly stiffer that normal appearing reference tissue, unlike gliomas which are soft, though not significantly. This can be attributed to the large range of stiffness variability found in FASIs, and their small nature making them more challenging to isolate. Similar to gliomas, FASIs have a significantly lower damping ratio than reference tissue. To our knowledge, this is the first time the mechanical properties of non-neoplastic FASIs in NF1 have ever been measured in vivo.

The mechanical underpinnings of FASI pathology are not well understood. FASIs show heterotopia, increased size and number of myelin vacuoles, gliosis, a buildup of intramyelinic edema, and macrocalcification foci [35]. Together these abnormal microstructural variations appear to have a combined biomechanical effect, and while these phenomena have not been individually assessed to determine their contributions to FASI mechanics, we can speculate on their individual contributions. MRE has shown that in hydrocephalus, which is characterized by a presence of unbounded fluid, brain tissue appears soft [36]. Conversely, bounded intramyelinic edema may be stiffening the individual myelin sheaths due to swelling, which in aggregate could make FASI areas stiffer. Macrocalcification foci refer to larger or more prominent accumulations of calcium; in most organs, calcium deposits lead to a macroscale stiffening of the tissue area [37]. Finally, gliosis refers to the proliferation and hypertrophy of glial cells, which occurs usually in response to inflammation; gliosis can form glial scars which are a dense network of astrocytic tissue [38]. However, increasing size and number of myelin vacuoles is likely to reduce mechanical integrity [39], and in some cases glial scars have shown to be mechanically soft [40]. Therefore, these contradictory processes make it challenging to elaborate on the expected directionality of mechanical properties of FASIs without more in vivo and ex vivo work.

This study had several limitations. Gliomas are heterogenous, and even with restricting our sample to LGGs, we observed large variability in tumor size, location, and cell type in our population. While we did not see evidence of trends in tumor mechanical properties based on these characteristics, they likely result in some variability not fully characterized. Histopathological diagnoses were not available for all the included tumors as some LGGs were never resected. Further, our population spans the pediatric age range from age four and older, and brain tissue changes significantly during maturation. To account for this, we used an individual’s normal appearing white matter as the reference, but future studies with larger samples sizes could more appropriately parse the interaction between maturation and tumor properties. Finally, image quality and resolution could have influenced our findings. We confirmed quality of the shear wave using the standard MRE metric octahedral-shear strain signal-to-noise ratio, but it is possible that other image artifacts could influence our data, including geometric distortion near high susceptibility regions [41]. We also acknowledge that the images were lower resolution than optimal for measuring the FASIs, which can be smaller than 1 mm. Because they are often clustered close together, we were able to treat them as a distinct unit, but their size created limitations for individual FASI analysis.

## Conclusion

Here we investigate the mechanical properties of pediatric central nervous system lesions including low-grade gliomas, which are softer and with lower viscosity than reference, and FASIs in NF1, which are stiffer and have lower damping ratio than reference. Pediatric gliomas are cellularly, molecularly, and cytoarchitecturally different than adult gliomas, and treatment has different implications for the developing brain than for the adult brain, therefore these pediatric tumors require separate considerations from adult tumors. While mechanical property analysis of both pediatric gliomas and FASIs is far from complete, this work provides an initial step in understanding how mechanical properties can be useful in clinical or surgical management of pediatric brain tumors.

## Data Availability

Anonymized data are available from the corresponding author on reasonable request.

## Abbreviations

FASI: Focal abnormal signal intensities
LGG: Low grade glioma
MRE: Magnetic resonance elastography
NF1: Neurofibromatosis type 1
NLI: Nonlinear inversion

## Declarations

### Presentation in part or whole at any meeting

International Society for Magnetic Resonance in Medicine, 2023 Annual Meeting, American Society of Pediatric Neuroradiology, 2023 Annual Meeting, American Society of Functional Neuroradiology 2023 Annual Meeting.
